# Modes of Action of Microbially-Produced Phytotoxins

**DOI:** 10.3390/toxins3081038

**Published:** 2011-08-22

**Authors:** Stephen O. Duke, Franck E. Dayan

**Affiliations:** United States Department of Agriculture, Agricultural Research Service, Natural Products Utilization Research Unit, P. O. Box 8048, MS 38677, USA; Email: Franck.Dayan@ars.usda.gov

**Keywords:** antibiotic, herbicide, phytotoxin

## Abstract

Some of the most potent phytotoxins are synthesized by microbes. A few of these share molecular target sites with some synthetic herbicides, but many microbial toxins have unique target sites with potential for exploitation by the herbicide industry. Compounds from both non-pathogenic and pathogenic microbes are discussed. Microbial phytotoxins with modes of action the same as those of commercial herbicides and those with novel modes of action of action are covered. Examples of the compounds discussed are tentoxin, AAL-toxin, auscaulitoxin aglycone, hydantocidin, thaxtomin, and tabtoxin.

## 1. Introduction

Microbes are a lucrative source of phytotoxins, e.g., [1,2,3,4,5,6,7,8,9,10]. The evolutionary pressure for phytotoxin production is obvious with microbial plant pathogens, but many non-pathogenic soil microbes also produce potent phytotoxins, and the role of these compounds in chemical ecology is less clear. An example of the latter case is the production of bialaphos by several *Streptomyces *species [10,11]. Most of the previous reviews of microbially-produced phytotoxins have focused on aspects of the compounds other than their modes of action. The reviews by Duke *et al.* [1] and Cutler *et al.* [12] are exceptions. Any review that focuses on mode of action leaves out many microbial phytotoxins for which we have little or no information on their molecular target site. We also exclude larger phytotoxic peptides (>10 amino acids). 

The mode of action facet of phytotoxins from microbes is overdue for an update, which we provide in this short review. We approach the topic from the standpoint of effects on general plant functions, with details about specific molecular target sites when they are available. 

## 2. Amino Acid Metabolism

### 2.1. Aminotransferases

**Figure 1:**
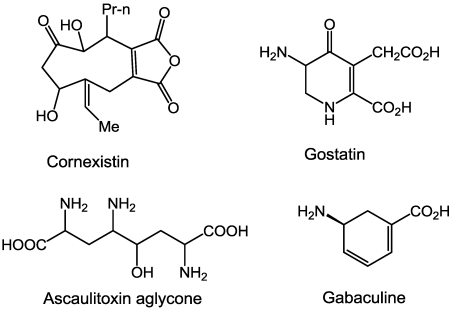
Structures of some microbial compounds known or thought to inhibit amino transferases.

Several microbial secondary compounds either inhibit an amino transferase or appear to have such a mode of action. Cornexistin (Figure 1), a fungal metabolite from *Paecilomyces variotii*, was patented as a herbicide. The amino transferase inhibitor aminooxyacetate causes identical herbicidal symptoms in duckweed [13]. Cornexistin inhibits aspartate amino transferase activity at high concentrations only after incubation in a plant cellular extract, suggesting that cornexistin is a proherbicide that must be metabolized to an amino transferase inhibitor. Gostatin (Figure 1), a product of *Streptomyces sumanensis* [14], is a potent amino transferase inhibitor that is phytotoxic [15].

Gabaculin (Figure 1), a product of *Streptomyces toyacaenis* [16], is an inhibitor of several aminotransferases e.g., [17]. In plants it strongly inhibits glutamate 1-semialdehyde aminotransferase, an enzyme required for 5-aminolevulinate synthesis and thus porphyrin and chlorophyll synthesis [16,18]. This compound will be discussed in more detail under section 11 on porphyrin synthesis.

Ascaulitoxin aglycone (Figure 1), a product of *Ascochyta caulina*, a fungus being studied as a potential mycoherbicide [19], is a potent phytotoxin that has profound effects on amino acid metabolism as determined by metabolic profiling [20]. Feeding treated plants with most amino acids reversed the effects of the toxin. However, *in vitro* assays found that the toxin did not inhibit alanine aminotransferase nor alanine:glyoxylate aminotransferase, leading the authors to speculate that it might inhibit another amino transferase or one or more amino acid transporters.

### 2.2. β-Cystathionase

**Figure 2:**
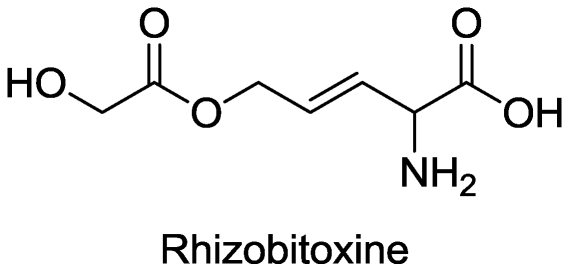
Structure of rhizobitoxine.

Rhizobitoxine (Figure 2) is a phytotoxin produced by some *Bradyrhizobium *strains [21]. It inhibits β-cystathionase, which is required for methionine synthesis [21,22]. This toxin is phytotoxic enough to have been considered as a commercial herbicide [23]. Since synthesis of the essential plant hormone ethylene is dependent on methionine, one could assume that ethylene synthesis would be greatly inhibited in plants treated with this compound. However, rhizobitoxine also directly inhibits production of ethylene from methionine [24] by inhibition of 1-aminocyclopropane-1-carboxylate synthase [25].

### 2.3. Glutamate Synthase

**Figure 3:**
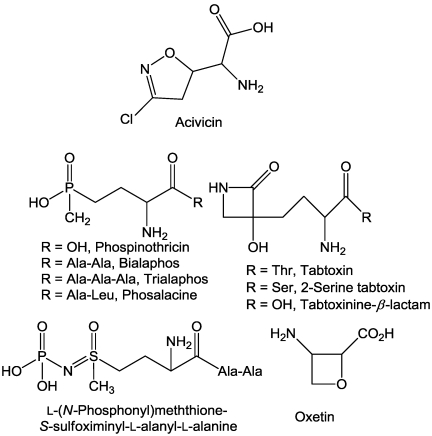
Structures of glutamate synthase and glutamine synthetase inhibitors from microbes.

Acivicin (Figure 3) is a product of *Streptomyces sviceu* [26] that has been patented as a herbicide [27]. It has not been well studied in plants, but has been well researched as a pharmaceutical. Acivicin is an analogue of glutamine and inhibits a number of glutamine-dependent enzymes, including glutamate synthase [28]. It also inhibits amidophosphoribosyltransferase, phosphoribosylformylglycinamidine synthase, GMP synthase, and γ-glutamyltranspeptidase [29,30,31]. Unfortunately, the effects of this toxin on these enzymes in plants are not published. 

### 2.4. Glutamine Synthetase

Phosphinothricin (Figure 3) and several other microbial products are inhibitors of glutamine synthetase (GS) [32]. This is perhaps the largest collection of microbial compounds that target a particular enzyme. Most of these compounds are of bacterial origin (from either *Pseudomonas syringae *plant pathovars or from soil-born *Streptomyces *species). These compounds are all analogues of glutamate, two of them are also produced from inactive di- or tripeptide protoxins (Figure 3). 

*Streptomyces hygroscopis *and *S. viridochromogenes *both produce bialaphos (Figure 3). This tripeptide does not inhibit GS, but must be metabolized in plants and microbes to L-phosphinothricin, the active GS inhibitor [33]. Inhibition of GS causes accumulation of toxic levels of ammonium, as well as a disruption of amino acid and other primary metabolism [32]. One of the earliest general physiological effects is cessation of photosynthesis [34]. Both bialaphos and phosphinothricin are sold as commercial herbicides. Trialaphos and phosalacine, produced by *S. hygroscopicus* sp. KSB-1285 and *Kitasatosporia phosalacinea*, respectively, also release phosphinothricin upon hydrolysis [35,36].

Bialaphos is produced by fermentation. It has a very small market as a herbicide in Japan. Phosphinothricin is sold as a synthetic mixture of L- and D-phosphinothricin sold under several trade names, but given the herbicide common name of glufosinate [37]. The D-isomer is inactive as a GS inhibitor. Glufosinate is one of the most successful commercial herbicides used throughout the world. Oxetin (Figure 3) from *Streptomyces *sp. OM-2317 [38] and the tripeptide L-(*N*^5^-phosphono)methionine-*S*-sulfoximinyl-L-alanyl-L-alanine from an unclassified strain of *Streptomyces* [39], are also GS inhibitors. Oxetin is a very weak GS inhibitor. The latter compound is inactive as the tripeptide, but degrades into two known strong GS inhibitors, phosphomethionine sulfoximine and methionine sufoximine. 

Several *Pseudomonas syringae* pathovars produce tabtoxin (Figure 3), a dipeptide prophytotoxin. Tabtoxin is not a GS inhibitor, but it is hydrolyzed *in planta* to form the potent GS inhibitor tabtoxinine-β-lactam [40,41]. Analogues of tabtoxin, such as 2-serine-tabtoxin [42], valyl-alanyl-tabtoxin, alanyl-tabtoxin, and alanyl-analyl-tabtoxin [43] have also been reported from various actinomycetes. 

### 2.5. Ornithine Transcarboxylase

**Figure 4:**
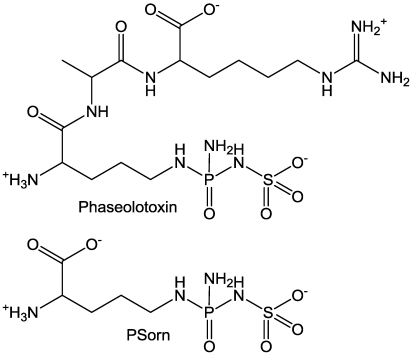
Phaseolotoxin and PSorn.

The product of ornithine transcarboxylase (OCTase) is citrulline, a precursor of arginine. So, inhibition of this enzyme results in loss of arginine production. Phaseolotoxin (Figure 4) is a tripeptide produced by *Pseudomonas syringae *pv. *phaseolicola. *Phaseolotoxin is a protoxin, in that peptidases of the plant must convert it to *N*^δ^*-(N*^1^*-*sulfodiaminophospinhyl)-L-ornithine (PSorn), which is a potent inhibitor of OCTase [44]. 

## 3. Cellulose Synthesis

**Figure 5:**
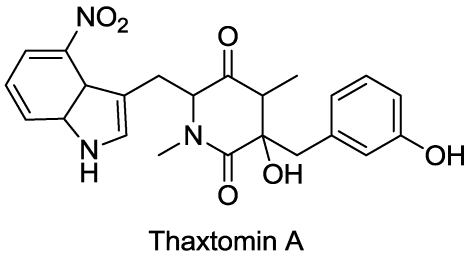
Structure of thaxtomin A.

Thaxtomin A (Figure 5) belongs to a group of cyclic dipeptides (2,5-diketopiperazines) which arise from the condensation of 4-nitrotrytophan and phenylalanine groups. Structure-activity studies determined that the presence of a 4-nitroindole group is necessary to maintain phytotoxicity of these metabolites [45]. These potent toxins are produced by several species of the gram-positive filamentous bacteria in the genus *Streptomyces* (e.g., *S. scabies* and *S. eubacteria*) that cause scab disease in potato and in several taproot crops.

Typical phenotypic responses of plants exposed to thaxtomin A include reduced seedling growth, cell swelling, and lignification of cell walls. Biochemically, thaxtomin inhibits cellulose synthesis. *Arabidopsis thaliana* seedlings treated with thaxtomin A have lower crystalline cellulose and higher content of pectins and hemicellulose in their cell wall, relative to untreated plants. This is accompanied by an alteration of the expression of genes involved in primary and secondary cellulose synthesis as well as genes associated with pectin metabolism and cell wall remodeling. Thaxtomin A affects the formation of the cellulose synthase complexes on the outside of the plasma membrane, leading to its dissociation from the cortical microtubule cytoskeleton [46].

## 4. Energy Transfer

**Figure 6:**
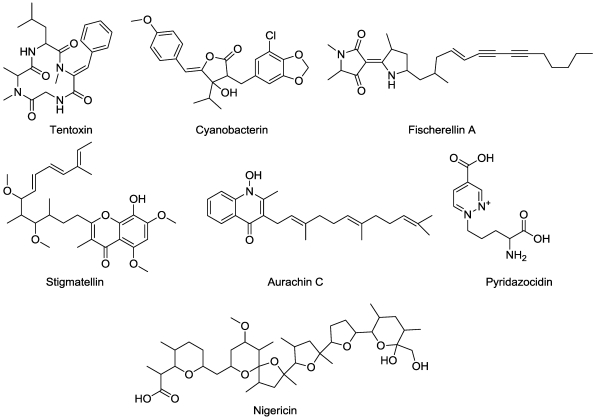
Microbially-derived phytotoxins that act on energy transfer functions.

Tentoxin (Figure 6), a cyclic tetrapeptide from the plant pathogen *Alternaria alternata*, inhibits chloroplast development, which phenotypically manifests itself as chlorotic tissue [47,48]. These papers indicate that there is no direct effect of tentoxin on chlorophyll synthesis. Two fundamental processes are linked with this phenotype. This first is inhibition of energy transfer of the chloroplast-localized CF_1_ ATPase [49,50]. One would think that this process alone could account for the chlorosis, but tentoxin also completely inhibits the transport of nuclear-coded enzyme polyphenol oxidase (PPO) into the plastid, even in etioplasts which should have no CF_1 _ATPase activity [51]. Without this processing, PPO has no enzyme activity. Inhibition of these two processes seems to be linked, in that both processes are inhibited *in vivo* in tentoxin-sensitive plant species and not affected in insensitive species [52]. Nevertheless, the coding of the β subunit of proton ATPase at codon 83 seems to account for susceptibility of plants to tentoxin [53]. Coding for glutamate at codon 83 correlates for resistance and aspartate coding results in susceptibility to tentoxin. Mutagenesis of *Chlamydomonas reinhardtii* to change gluamate to aspartate resulted in a change from resistant to susceptible. Later, tentoxin was suggested to exert its effect on chlorophyll accumulation through overenergization of thylakoids [54], but this does not explain the profound effects of the compound on PPO processing in etioplasts without thylakoid membranes. The linkage of the β subunit of proton ATPase to PPO processing remains to be explained. Understanding this relationship may help to explain the role of PPO in the plastid, where enzymatic activity is latent [55,56]. The true physiological role of PPO in a functional chloroplast is still a mystery.

Nigericin (Figure 6), a product of *Streptomyces hygroscopicus*, is an uncoupler of photophosphorylation [57]. It inhibits photosynthesis with decreased ATP/ADP ratios, decreased energy quenching, and hyper-reduction of Q_A_ [58]. 

Several microbial phytotoxins inhibit photosynthetic electron transport. These include cyanobacterin, fischerellin A, stigmatellin, and the aurachins (Figure 6). The first two of these compounds are produced by cyanobacteria. Cyanobacterin is a halogenated compound from the freshwater cyanobacterium *Scytonema hofmanni *that inhibits electron transport of photosystem II [59]. Fischerellin from the cyanobacterium *Fischerella muscicola *produces fischerellin A that inhibits PSII of green algae and higher plants [60]. Stigmatellin, produced by the myxobacterium *Stigmatella aurantica*, inhibits photosynthetic electron transport at both the D-1 site of synthetic photosynthetic inhibitors and at the cytochrome b6/f-complex [61]. The aurachins, a group of quinoline compounds from *Stigmatella aurantica*, also inhibit photosynthesis at the same two sites as stigmatellin [62]. Pyridazocidin (Figure 6), a cationic compound from soil *Streptomyces* species, causes rapid plant necrosis and chlorosis, much like that of bipyridinium herbicides like paraquat [63]. Studies with isolated chloroplasts showed that its mode of action is exactly like bipyridiniums, diverting electrons from photosystem I to become reduced to a reactive radicle that subsequently generates superoxide radicle, resulting in a cascade of destructive oxidative processes. This is the only natural phytotoxin of which we are aware with this mode of action.

## 5. Jasmonic Acid Analogues

**Figure 7:**
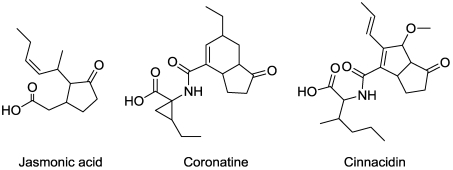
Jasmonic acid and phytotoxin analogs.

Jasmonic acid (Figure 7) is a plant hormone derived from linolenic acid. It plays a major role in regulating growth and development, as well as responses to both abiotic and biotic stress. Coronatine (Figure 7) is a jasmonate analog produced by *Pseudomonas coronafacience *[64]. It usurps jasmonate-controlled signaling pathways [65], thereby deregulating many essential processes. The typical symptom of this toxin is chlorosis of developing tissues. Cinnacidin (Figure 7), a product of the fungus *Nectria *sp. DA060097, has a similar mode of action to coronatine [66]. 

## 6. Lipid Metabolism

**Figure 8:**
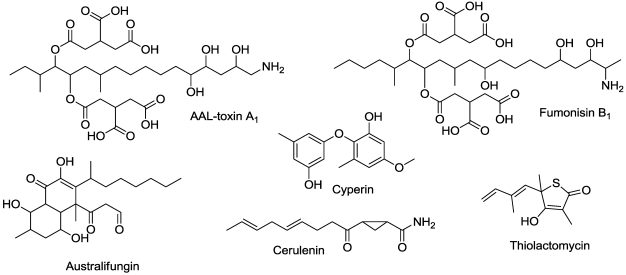
Microbial compounds that affect lipid synthesis.

A series of structurally related fungal metabolites specifically inhibit ceramide synthase (sphinganine-*N*-acyltransferase) in plants. These include several analogues of AAL toxin and fumonisin [67,68,69,70] (Figure 8). AAL toxins are produced by *Alternaria alternata* tomato pathovars and fumonisins are produced by *Fusarium* spp. AAL toxins were originally reported to be host specific, but they are phytotoxic to many plant species, as are their close structural analogues, the fumonisins. These compounds are analogues of the substrate for ceramide synthase, although australifungin is only a weak analog (Figure 8) [70]. When plant tissue is treated with these inhibitors, the sphingolipid precursors and precursor derivative levels are rapidly elevated to concentrations many fold more than found in untreated tissues [71]. This precedes rapid loss of plasma membrane integrity. Others have sought to explain the action of this family of toxins by invoking induction of apoptosis (programmed cell death) [72,73], but the effects are so rapid at even low doses, that this phenomenon seems unlikely to play a direct role except at very low doses. Treatment of plants with the sphingoid base precursors of ceramide synthase causes similar effects to those caused by the inhibitors of ceramide synthase [74]. They cause rapid, light-independent cellular leakage through dysfunction of the plasma membrane. Sphingoid bases also cause generation of reactive oxygen species (ROS) [75] in plant cells. Rapid formation of ROS in the plasma membrane can cause cell death unrelated to apoptosis, whereas slower formation can cause programmed cell death. 

Thiolactomycin (Figure 8) is produced by unidentified species of *Norcardia* and *Streptomyces *and is an inhibitor of both plant and animal type II dissociated fatty acid synthetase [76]. It is a very potent inhibitor of incorporation of acetate into fatty acids of chloroplasts [77]. Cerulenin (Figure 8), a product of the fungus *Cephalosporium cerulens*, inhibits de novo fatty acid synthesis in plastids [78]. Like thiolactomycin, it is an inhibitor of fatty acid synthetases, but it is not as active as an inhibitor [79]. 

The diphenyl ether compound cyperin (Figure 8), a metabolite of *Preussia fleischhakii*, *Phoma sorghina*, and *Ascochyta cypericola *[80,81,82], inhibits plant enoyl (acyl carrier protein) reductase (ENR), which is the target site of a synthetic diphenyl ether called triclosan. Inhibition of ENR results in light-independent disruption of membrane integrity [83].

## 7. Membrane Function

**Figure 9:**
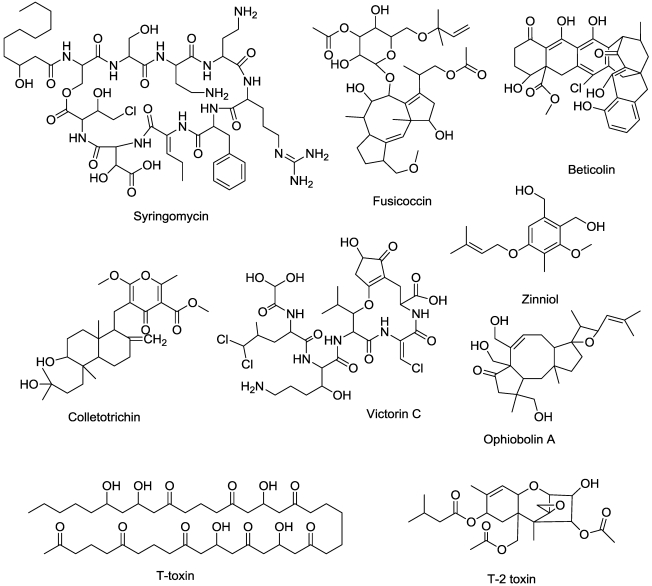
Microbially-produced compounds that affect membrane function.

Syringomycin (Figure 9), from *Pseudomonas syringae*, is one of the many cyclic lipodepsinonapeptide microbial phytotoxins. Structurally related compounds from the same organism with similar modes of action are syringotoxin and syringostatins [84,85]. These compounds are large molecules that typically have a polar peptide head and a hydrophobic 3-hydroxy fatty acid tail. 

This hydrophobic tail of varying length (from C10 to C14) is bound to the *N*-terminal serine residue via an amide bond. The *macrocyclic* lactone ring is obtained via an ester linkage to the *C*-terminal 4-chlorothreonine. Syringomycin often contains uncommon amino acids such as 2,3-dehydroaminobutyric acid, 3-hydroxyaspartic acid, and 4-chlorothreonine, as well as serine D-isomers and 2,4-diaminobutyric acid [86]. Structure-activity relationship studies reported that chlorination of the molecule is important for biological activity. 

Syringomycin induces rapid necrosis in plant tissues by forming pores that are freely permeable to cations (e.g., K^+^, H^+^, and Ca^2+^) within the plasma membrane. Nanomolar amounts of syringomycin are sufficient to induce loss of membrane integrity and cell death [87].

The beticolins (Figure 9), a yellow group of toxins from *Cercospora beticola*, self assemble into multimeric ion channels that disrupt membrane function [88,89]. T-toxins (Figure 9) are host-specific, trichothecene phytotoxins from the fungi *Cochiobolus heterstrophus*,* Phyllostica maydis*, and *Bipolaris maydis. *They inhibit mitochondrial respiration by binding an inner mitochondrial membrane protein in sensitive plants, resulting in pore formation, leakage of NAD^+^, and other ions, as well as subsequent mitrochondrial swelling [90,91]. Fusicoccin (Figure 9) a product of the fungus *Fusicoccum *(*Phomopsis*) *amygdali *irreversibly activates the plant plasma membrane H^+^-ATPase, leading to inability of stomata to close and subsequent lethal wilting [92,93].

Victorin C (Figure 9), a fungal product of *Cochiobolus victoriae*, induces a collapse of the mitochondrial transmembrane potential, which results in a mitochondrial membrane transition [94]. It also binds the P protein of the glycine decarboxylase complex of the mitochrondria [95]. All of this has been associated with programmed cell death, but it may also act at the cell surface to cause a hypersensitive response via plasma membrane ion fluxes [95].

Colletotrichin (Figure 9) is a highly phytotoxic compound from several *Colletotrichum* species, e.g., [96]. Ultrastructurally, the first effect of this compound is disintegration of the plasma membrane, accompanied by massive cellular leakage [97]. The effect is not light dependent and could not be reversed with antioxidants, suggesting that it has a direct effect on the plasma membrane. 

Nigericin (Figure 6), a *Streptomyces hygroscopicus* product is a phytotoxic postassium ionophore [98]. Zinniol (Figure 9), a product of several *Alternaria* species and one *Phoma *species, binds plant protoplasts and stimulates Ca^++^ entry into cells [99]. It may act on a specific class of plant calcium channel. There are a number of other compounds produced by plant pathogens that are structurally related to zinniol, but their mode of action has not been determined.

T-2 toxin is a trichothecene that, unlike the other trichothecenes that inhibit protein synthesis, also causes plant plasma membrane leakage of electrolytes at low concentrations [100].

Ophiobolins (Figure 9), tricyclic sesquiterpene phytotoxins from certain species of *Bipolaris* and other fungal genera, cause many symptoms on plants that were considered to be largely due to effects on the plasma membrane [101]. It effects on maize root ion leakage correlate well with its direct antagonism of calmodulin [102]. Its effects on calmodulin cause inhibition of transport of nuclear-coded proteins into both the mitochondrion [103] and the plastid [104].

## 8. Mitotic Disruptors

**Figure 10:**
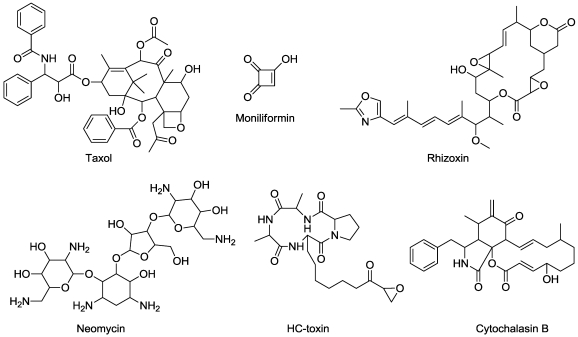
Structures of some microbially produced plant mitosis disruptors.

Numerous natural products inhibit plant cell mitosis rather directly by interfering with the function of microtubules. However, most all of these are products of plants (e.g., colchicine) [105]. Taxol (Figure 10), a potent mitotic inhibitor, first found in yew (*Taxus*) species, has subsequently been found to be produced by several endophytic fungi, e.g., [106,107]. In addition to being a potent toxin for mammalian cancer cells, taxol is an effective inhibitor of plant cell mitosis [108]. In both cases it hyperstablizes microtubules, preventing the cycling of tubulin subunits required for microtubule function [109].

Rhizoxin (Figure 10), a product of a bacterial endosymbiont of the plant pathogen *Rhizopus microsporus*, binds to β-tubulin, thereby inhibiting microtubule formation [110,111]. These findings were the first reported case of a phytotoxin from a fungus being derived from a bacterial endosymbiont. The producing fungus has a rhizoxin-resistant form of β-tubulin [112]. Neomycin (Figure 10), an aminoglycoside antibiotic from *Streptomyces fradiae*, disrupts mitosis in plant cells [113,114]. It does this by inhibiting polyphosphoinositide cycling through inhibition of hydrolysis of phosphatidylinositol 4,5-bisphosphate into inositol 1,4,5-triphosphate and 1,2-diacylglycerol. This apparently is the mechanism of phytotoxicity in both higher plants and algae [115]. 

Moniliformin (Figure 10), a mycotoxin from *Fusarium moniliforme*, is phytotoxic and arrests mitosis of maize root meristematic cells at the metaphase stage [116]. The mitotic spindle was disrupted, but no direct effect on tubulin has been observed.

Functional actin filaments are required for normal mitosis, as well as other cell functions related to the cell cytoskeleton. Cytochalasins (A-H) (Figure 10) are actin-binding metabolites of several fungal species, such as *Phoma exigua* and *Zygosporium masonii* [117]. Binding actin prevents actin polymerization into filaments, thus inhibiting the processes that require actin filaments, such as mitosis and other plant processes [12,118] 

HC-toxin (Figure 10), a cyclic tetrapeptide from the maize pathogen *Cochliobolus carbonum*, inhibits growth and cell division of target plants [119]. Its molecular site of action is histone deacetylase (HDAC). Histones associated with chromosomal DNA become hyperacetylated in treated plants. This condition apparently prevents cell division. HC-toxin may also significantly alter gene expression in ways that would be detrimental to the plant. HC-toxin inhibits this enzyme in all plants and animals and is the basis for new anti-cancer drugs. A number of related fungal compounds are all known or presumed HDAC inhibitors. 

## 9. Nucleic Acid Synthesis

**Figure 11:**
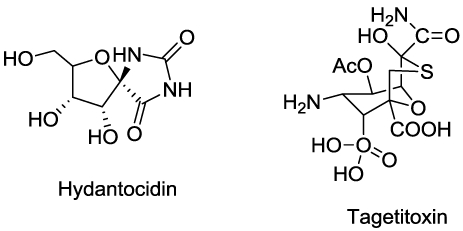
Microbial compounds that inhibit nucleic acid synthesis.

Tagetitoxin (Figure 11) from a pathovar of *Pseudomonas syringae* inhibits plastid RNA polymerase [120]. This results in a yellow, chlorotic phenotype. It also inibibits RNA polymerase III from animals [121]. Its inhibition is characterized by stalling the elongation complex at several points in the template that are template-dependent [122]. 

Hydantocidin (Figure 11), a spironucleoside from *Streptomyces hygroscopis*, is highly phytotoxic [123]. Hydantocidin and a number of synthetic analogues have been patented as herbicides. It is phosphorylated *in vivo*, and the derivative, 5'-phosphohydantocidin (5PH), inhibits adenylosuccinate synthetase (ASS) [124,125,126,127], an enzyme required for purine synthesis. ASS converts IMP to AMP. 5PH inhibits ASS by competitively inhibiting it through binding the IMP substrate binding site, forming a dead-end complex [128]. ASS is also inhibited by ribofuranosyl triazolone, a phytotoxic product of an *Actinomadura* species [129]. It is a broad spectrum herbicide in greenhouse studies. Guanine monophosphate synthetase, (GMP synthase) converts xanthosine monophosphate to guanosine monophosphate. As mentioned in the amino acid metabolism section, acivicin (Figure 3) is an inhibitor of this enzyme [30].

## 10. Photodynamic Compounds

**Figure 12:**
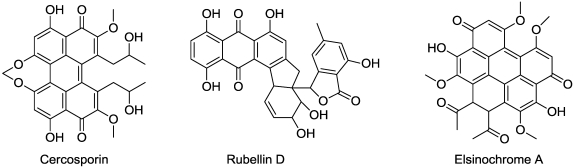
Structures of some photodynamic microbial phytotoxins.

Cercosporin (Figure 12) is a red fungal toxin that was first isolated in the 1950s from species of the fungal genus *Cercospora*. This photodynamic pigment is a potent photosensitizer and, in the presence of light and oxygen, it generates singlet oxygen (^1^O_2_) and superoxide (O^-•^_2_) ions that induce rapid membrane peroxidation and cellular death [130]. Isocercosporin from *Scolecotrichum gramminis *is also photodynamic [131]. Elsinochromes (Figure 12) from the fungus *Elsinoe fawcetti* are red pigments of very similar structure to cercosporin [132]. There are several other fungal perylenequinone phytotoxins [133]. They have the same mode of action as cercosporin.

Cercosporin is a general toxin that will affect the lipid bilayers of any cells including plants, animals, bacteria, and fungi. This compound may also have antiviral activity and inhibit protein kinase C. In plants, tissues and cells treated with cercosporin incur rapid, light-dependent damage to membranes, which is accompanied with an elevation of lipid peroxidation products [134].

Rubellin D (Figure 12), from the fungus *Ramularia collo-cygni*, is also a phytodynamic pigment that is light-dependent for its activity [135]. This anthraquinone derivative causes singlet oxygen-mediated α-linoleic acid peroxidation when exposed to light. 

## 11. Porphyrin Synthesis

Cyperin (Figure 8) is a natural diphenyl ether phytotoxin produced by several fungal plant pathogens mentioned in Section 6 [80,81,82]. At high concentrations, this metabolite inhibits protoporphyrinogen oxidase, a key enzyme in porphyrin synthesis [136]. However, unlike synthetic herbicidal diphenyl ethers that target this enzyme, the mode of action of cyperin is light-independent, causing membrane degradation in the dark. Its main effect as a herbicide is on plant enoyl (acyl carrier protein) reductase (discussed in Section 6).

Gabaculine (Figure 1) is a strong inhibitor of the enzyme glutamate-1-semialdehyde aminotransferase, an enzyme involved in the early porphyrin pathway [137,138]. Inhibition of this enzyme results in stopping synthesis of 5-aminolevulinic acid. By inhibiting porphyrin synthesis, it inhibits both heme and chlorophyll synthesis [139,140], as well as that of the tetrapyrrole phytochrome [141]. 

## 12. Protein Synthesis

**Figure 13:**
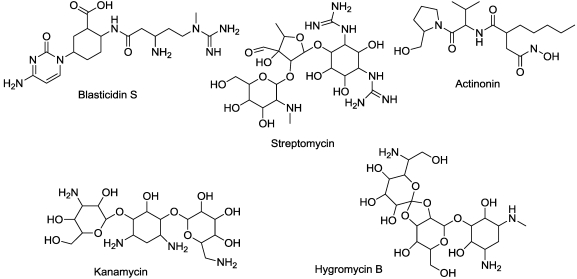
Microbial phytotoxins that inhibit protein synthesis.

In addition to being a protein synthesis inhibitor of bacteria, the antibiotic streptomycin (Figure 13) inhibits protein synthesis of plastids [142]. It binds to 30S ribosomal subunits to cause this effect [143]. Kanamycin and hygromycin (Figure 13), aminoglycoside antibiotics from *Streptomyces *species, are both phytotoxic [144,145]. They both inhibit protein synthesis by interaction with ribosomes, although kanamycin inhibits prokaryotic-type protein synthesis, while hygromycin inhibits both prokaryotic and eukaryotic protein synthesis [146]. 

Actinonin (Figure 13), a product of an *Actinomyces* MG848-hF6 [147], inhibits plastid peptide deformylase (DEF), an enzyme required for *N*-terminal protein processing of plastid-encoded proteins [148,149]. This compound is a non-selective herbicide that results in chlorotic plants. Overexpression of two of three different plant DEFs leads to resistance to actinonin [148,149,150].

The trichothecenes (see Figure 9 for an example of T-2 toxin), a large class of fungi-produced sesquiterpene mycotoxins, exert most of their effects by inhibiting protein synthesis [12]. They do this apparently by targeting the peptidyltransferase center of mitochondrial ribosomes [151]. One would expect that they would have the same effect on mitochondrial and perhaps plastid ribosomes. Indeed, transgenic modification of wheat with a trichothecene-resistant mitochondrial ribosome subunit, imparts partial resistance to a trichothecene-producing pathogen [152]. Most of the trichothecenes are produced by plant pathogens, including species from genera such as *Fusarium*,* Myrothecium*,* Trichoderma*, and *Cephalosporium. *

Blasticidin S (Figure 13) is a nucleoside antibiotic that is produced by several *Streptomyces *species, e.g., [153,154]. Blastocidin S is more phytotoxic to dicotyledonous than monocotyledonous species [155]. For example, protein synthesis is more affected in carrot than in rice. It inhibits translation of both eukaryotic and prokaryotic cells by inhibition of peptide bond formation by the ribosome through inhibition of peptidyl transferase [156,157,158].

## 13. Protein Binding

**Figure 14:**
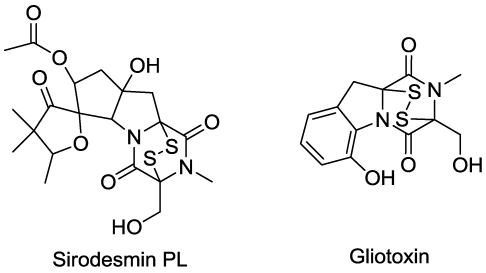
Microbial phytotoxins that directly bind proteins.

Compounds with internal disulfide bridges can covalently bind proteins, sometimes inactivating the protein function. They accomplish this by reaction of the disulfide bond with the cysteine components of proteins. Some fungal phytotoxins such as sirodesmin PL (Figure 14) from *Leptosphaeria maculans* and gliotoxin (Figure 14) have such internal disulfide bridges that conjugate proteins [159,160,161]. These compounds are also implicated in generation of reactive oxygen species by redox cycling [160]. Such compounds are generally broadly cytotoxic. 

## 14. Sugar Metabolism

**Figure 15:**
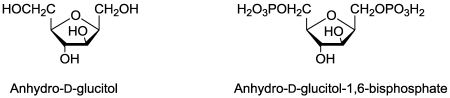
The inactive (left) and activated (right) forms of anhydro-D-glucitol.

Anhydro-D-glucitol (Figure 15), produced by the plant pathogenic fungus *Fusarium solani*, is mildly phytotoxic [162]. When phosphorylated by the plant (Figure 14), it is a close analog of fructose-1,6-bisphosphate, thereby inhibiting fructose-1,6-bisphophate aldolase activity, which is required for production of glyceraldehyde-3-phosphate and dihyroxyacetonephosphate in glycolysis [163]. 

## 15. Terpenoid Synthesis

**Figure 16:**
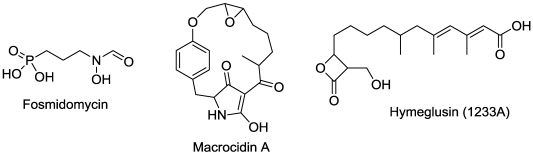
Microbial phytotoxins that inhibit terpenoid synthesis.

The macrocidins (Figure 16) from *Phoma macrostoma* are cyclic tetramic acids. Tetramic acid is an inhibitor of hydoxyphenylpyrutvate dioxygenase (HPPD), but the macrocidins appear to inhibit carotenoid synthesis by a different mode of action [164,165]. HPPD activity is required to produce the cofactor, plastoquinone, of phytoene desaturase, an enzyme involved in carotenoid biosynthesis. 

Fosmidomycin (Figure 16), a product of *Streptomyces lavendulae* [166], is highly phytotoxic, causing extreme chlorosis by inhibition of the non-mevalonic acid pathway, upon which production of most of the critical plant terpenoids depend [167,168]. Its enzyme target site is 1-deoxy-D-xylulose 5-phosphate reductoisomerase, an early enzyme in the pathway. 

Hymeglusin (Figure 16), also known as 1233A and L-659699, is a phytotoxin produced by several fungal plant pathogens [169,170]. It inhibits 3-hydroxy-3-methylglutaryl coenzyme A synthase of plants and animals [170,171]. This enzyme is required for synthesis of certain terpenoids (e.g., sterols) of the mevalonic acid pathway in plants and cholesterol in animals. 

## 16. Conclusions

This brief coverage should provide an appreciation for the amazing breadth of microbial phytotoxin structures and modes of action. The number of potential useable herbicide target sites has been a matter of concern among companies involved in herbicide discovery. Molecular methods to discover new target sites have not been particularly fruitful [172]. There are only about twenty molecular sites targeted by the hundreds of commercial herbicide active ingredients, and the last major target site was introduced to the marketplace over twenty years ago. However, it is clear from the many target sites of microbial phytotoxins, that nature has discovered many ways to kill a plant. The growing evolution of weed resistance to existing commercial herbicides has generated a new sense of urgency to discover and develop herbicides with new modes of action [173]. Many of the compounds mentioned in this review have been studied as potential templates for new herbicides with new modes of action. We expect that the growing need for new modes of action will generate a stronger interest in the use of microbial phytotoxins to discover new herbicide target sites.
