# Correction: Duke, S.O., *et al.*, Modes of Action of Microbially-Produced Phytotoxins. *Toxins* 2011, *3*,1038-1064

**DOI:** 10.3390/toxins4100955

**Published:** 2012-10-23

**Authors:** Stephen O. Duke, Franck E. Dayan

**Affiliations:** United States Department of Agriculture, Agricultural Research Service, Natural Products Utilization Research Unit, P. O. Box 8048, MS 38677, USA; Email: Franck.Dayan@ars.usda.gov

The authors are sorry to report that the structure of rhizobitoxine in Figure 2 in their published paper [1] was incorrect. The correct figure should be:

**Figure 2:**
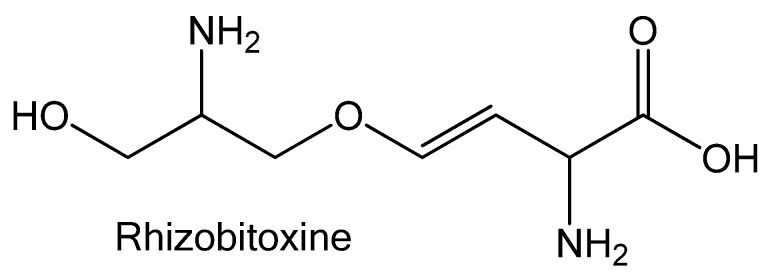
Structure of rhizobitoxine.

We apologize for any inconvenience caused to the readers.
